# Supplementary material for: Mitochondrial and Endoplasmic Reticulum Alterations in a Case of Amyotrophic Lateral Sclerosis Caused by TDP-43 A382T Mutation
Source: Int J Mol Sci. 2022 Oct 6;23(19):11881. doi: 10.3390/ijms231911881 (PMC9570187; doi:10.3390/ijms231911881)
Supplement: Supplementary file 1 [file ijms-23-11881-s001.zip › Supplementary figure S1.pptx]

## Slide 1
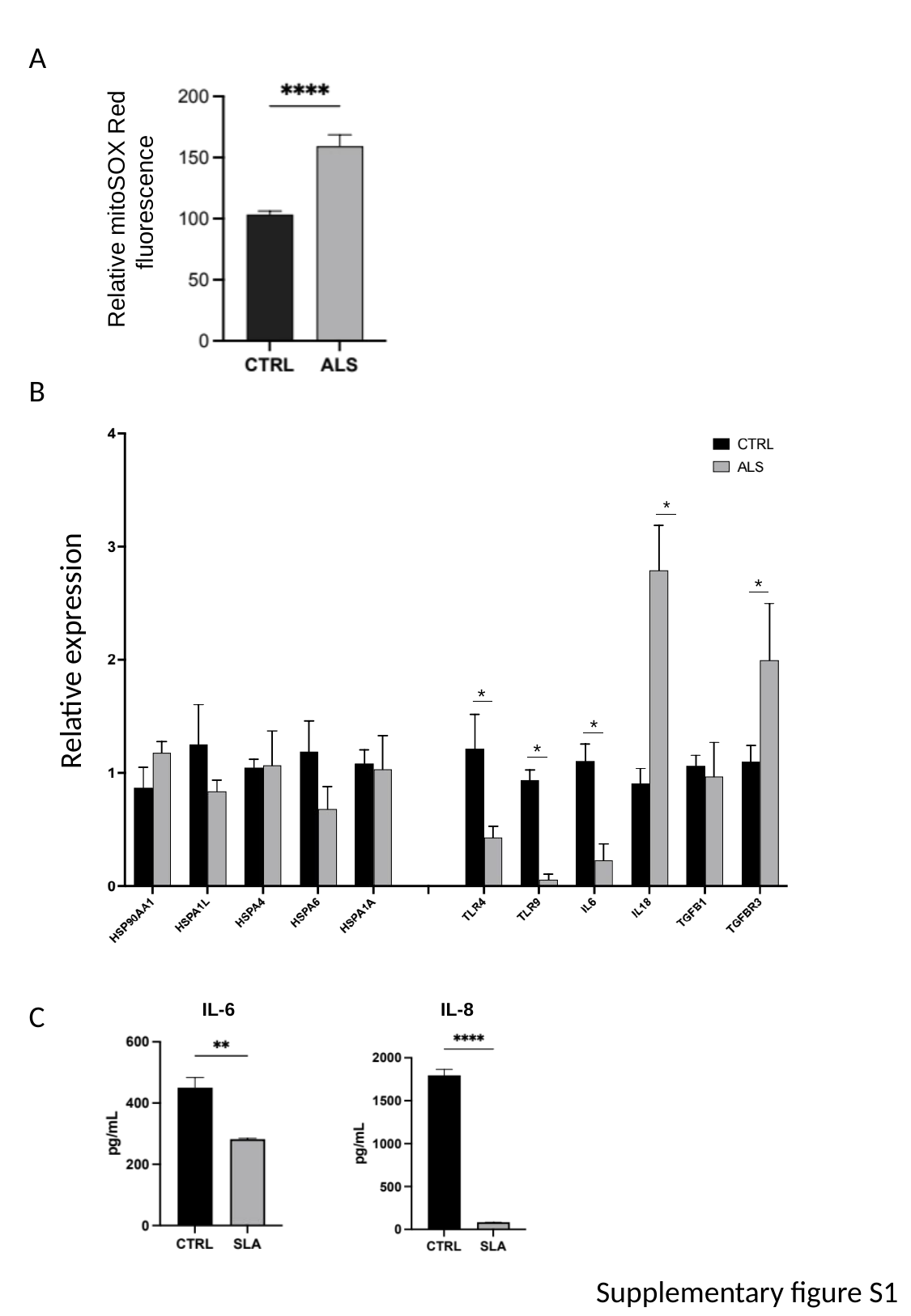

A
Relative mitoSOX Red
fluorescence
B
*
*
Relative expression
*
*
*
C
IL-6
IL-8
Supplementary figure S1
